# Relationships among self-study ability, critical thinking ability, cooperative ability, and problem-solving ability in Chinese undergraduate nursing students: an analysis of a longitudinal cohort via cross-lagged models

**DOI:** 10.3389/fpsyg.2025.1606156

**Published:** 2025-07-02

**Authors:** Dun Liu, Jianbin Lin, Jincheng Zhang, Xinchu Luo

**Affiliations:** ^1^The School of Nursing, Fujian Medical University, Fuzhou, Fujian, China; ^2^Fujian Health College, Fuzhou, Fujian, China

**Keywords:** self-study ability, critical thinking ability, collaborative ability, problem-solving ability, undergraduate students, nursing

## Abstract

**Background:**

There is currently a lack of research on the change in self-study ability, critical thinking ability, collaborative ability, and problem-solving ability in undergraduate nursing students before and after completing professional courses with blending teaching, as well as the mutual influence of different abilities.

**Objective:**

To understand the longitudinal and bidirectional relationships among self-study ability, critical thinking ability, collaborative ability, and problem-solving ability in undergraduate nursing students.

**Method:**

A longitudinal study design was implemented, incorporating two distinct temporal measurement points: baseline assessment prior to the commencement of professional nursing courses and subsequent evaluation following course completion. Correlation analysis and a cross-lagged model were used to explore the changes in various abilities of undergraduate nursing students before and after completing professional courses, as well as the interactions between different abilities.

**Result:**

Problem-solving skills were correlated with various other variables. Self-directed learning ability at the initial time point was negatively correlated with certain variables. Self-study ability had a significant and negative predictive effect from Time 1 to Time 2. Collaborative ability significantly and positively influenced self-study ability from Time 1 to Time 2. Problem-solving ability showed a significant and positive predictive effect from Time 1 to Time 2 and positively influenced collaborative ability from Time 1 to Time 2. Self-study ability had a significant and negative impact on critical thinking from Time 1 to Time 2.

**Conclusion:**

Undergraduate Nursing students' self-study, collaborative, and problem-solving abilities are interrelated. Beginning in the early years of study, educators should revamp teaching methods and models to assist students in unlearning conventional learning and thought processes. Teachers should aim to foster collaborative problem-solving skills by applying knowledge, thereby augmenting students' overall problem-solving proficiency.

## 1 Introduction

Nursing, as a first-level discipline in the medical field, is a discipline and profession centered on human health, with the goal of providing personalized and precise management and health education. The professional abilities, learning abilities, and thinking abilities of undergraduate nursing students, who are the backbone of future clinical nursing work, directly affect the innovation level, disciplinary development, and overall quality improvement of clinical nursing staff in China (Wang et al., 2019). In the context of new medical science construction, the internet, the internet of things, artificial intelligence, big data, and genomics further expand the knowledge and extend the reach of nurses; therefore, nurses must stay up-to-date with the times and learn to remotely, accurately and dynamically monitor patients' health status, lifestyle, disease progress, treatment compliance, etc. to provide personalized and accurate treatment anytime and anywhere (Lin et al., 2020). Therefore, there is an urgent need to train many professional and practical nursing professionals with good comprehensive abilities and qualities to provide the appropriate conditions in clinical work for achieving personalized and precise health management and guidance.

The core competencies of clinical nursing staff include self-study, critical thinking, collaborative, and problem-solving abilities. Self-study ability refers to the ability to use metacognition and objective human and material resources to acquire and master the necessary knowledge and skills to provide high-quality nursing services. The constituent elements of self-study ability include self-management ability, information acquisition ability, and collaborative ability (Kundu, 2020). One of the training objectives of undergraduate nursing education in China at present is to train nursing professionals with strong self-study and scientific thinking abilities (Zhu et al., 2022). Both domestically and internationally, nursing educators are gradually emphasizing the cultivation of undergraduate nursing students' self-study abilities and incorporating them into the teaching objectives of undergraduate nursing programs. Critical thinking is a purposeful, meaningful, and self-regulated process of judgement and reflection (Lamont, 2020). In the process of training and educating undergraduate nursing students, critical thinking, which involves self-regulation and deep reflection in both academic and practical aspects, is essential. Critical thinking can serve as an effective basis for judging whether undergraduate nursing education meets quality standards (Lee and Oh, 2020). Cooperative ability, as a core professional skill, refers to the ability to establish good cooperative relationships with others, organize tasks in practice, plan task division, and ultimately achieve common goals (Tang et al., 2022). Collaborative ability is an effective way to increase the sense of responsibility among nursing students (Bin, 2018). In the process of learning professional nursing knowledge, working together to manage the relationships between individuals and teams and solve problems can achieve twice the result with half the effort. Therefore, for nursing students, collaborative ability is an essential evaluation indicator. Social problem-solving ability is defined as an individual's ability to identify specific problems encountered in daily life and discover effective solutions (Gu et al., 2022), which involves various aspects of human cognition, thought, emotion, behavior, etc. (Pertz et al., 2022). Problem-solving and social problem-solving abilities are essential skills for the nursing profession and have a significant impact on nurses' psychological resilience and various skills (Abdollahi et al., 2018). The cultivation of nurses' problem-solving ability needs to start in the nursing student stage, and the earlier the implementation is, the better the effect (Luo and Li, 2020). Therefore, self-study, critical thinking, collaborative, and problem-solving abilities are important training objectives and evaluation indicators in nursing education.

At present, in China, undergraduate nursing programs mostly have duration of 4 years. Students usually complete basic medical and professional courses in the first and second years and begin professional courses in the third year. The completion of nursing courses is an important part of cultivating the comprehensive abilities of undergraduate nursing students. In China, undergraduate nursing courses mainly include core courses such as nursing fundamentals, internal medicine nursing, and surgical nursing. These courses focus on human health, are closely related to clinical practice, are guided by modern medical models and holistic nursing concepts, and are framed by nursing procedures. They emphasize the cultivation of comprehensive abilities such as holistic nursing, humanistic care, critical thinking, and the ability to identify, analyze, and solve problems (Liu et al., 2021). Therefore, the objective of nursing courses is not only to gain professional knowledge but also, more importantly, to cultivate the comprehensive abilities of undergraduate nursing students. The teaching quality and effectiveness of undergraduate nursing courses greatly affect the quality of nursing talent and the sustainable development of clinical nursing (Kobayashi and Saeki, 2025).

In addition, in the process of learning in nursing courses, the interactions among self-study, critical thinking, collaborative, and problem-solving abilities may be bidirectional. In the process of learning in professional courses, students need to apply critical thinking skills to better grasp knowledge through self-study. Moreover, in the case of professional courses, it is necessary to apply collaborative and problem-solving skills to improve learning effectiveness, and in the process of collaboration and problem-solving, critical thinking skills must be applied, which further enhances self-study ability. The relevant theoretical framework also supports the correlations among these variables. For instance, social constructivist learning theory posits that knowledge is constructed through social interaction and collaboration, emphasizing the role of learners' active participation and teamwork in cognitive development. Specifically, collaborative skills (such as team discussions and role division) can facilitate knowledge sharing and the integration of diverse perspectives, thereby enhancing self-directed learning and problem-solving abilities. Additionally, this theory highlights the ideological influences in knowledge construction (e.g., the limitations of traditional nursing education models) and encourages learners to develop self-directed learning abilities through critical reflection, enabling individuals to better achieve knowledge construction (Epp et al., 2021). Thus, collaborative skills can promote the development of students' self-directed learning abilities, while critical thinking serves as an essential tool for reaching consensus during collaborative learning. Furthermore, systems thinking theory suggests that solving complex problems require holistic and dynamic analytical skills. Critical thinking lies at the core of systems analysis, and collaborative skills help integrate different perspectives to enhance overall capabilities. Self-directed learning provides the knowledge foundation for system optimization, aiding individuals in achieving dynamic analytical abilities (Dominici, 2017). Our preliminary research also indicates that nursing students' self-directed learning abilities can influence the development of critical thinking skills (Liu et al., 2021). Therefore, integrating theoretical insights with empirical findings suggests that nursing students' self-directed learning abilities can provide the knowledge reserve and exploratory motivation for critical thinking and problem-solving. Critical thinking optimizes collaborative strategies and problem-solving pathways through logical analysis and reflection, while collaborative skills improve team problem-solving efficiency through resource integration and conflict management, simultaneously strengthening individuals' self-directed learning motivation. There exists a strong correlation among critical thinking, self-directed learning, collaborative skills, and problem-solving abilities.

However, there is currently a lack of research on the change in the self-study, critical thinking, collaborative, and problem-solving abilities of undergraduate nursing students before and after completing professional courses with blending teaching, as well as the mutual influence of different abilities. To elucidate the longitudinal and bidirectional associations between self-directed learning, critical thinking, collaborative skills, and problem-solving abilities, this study employed a longitudinal research design. Data were collected at two distinct time points—prior to and following the completion of professional nursing courses—from undergraduate nursing students enrolled at a medical university in southeastern China. The changes in the various abilities of undergraduate nursing students, as well as the interactions among different abilities, were explored before and after the completion of professional courses, providing a basis for teaching reform in nursing professional courses.

## 2 Methods

### 2.1 Study design

In the present investigation, we implemented a longitudinal research design to systematically examine undergraduate nursing students. According to the training plan of the school, undergraduate nursing students complete basic medical courses in the first semester of their first year and complete professional nursing courses in the second semester of their junior year. Therefore, the first time point of this study was at the end of the first semester of the students' first year, and the second time point was the end of the second semester of the students' junior year. The first survey was conducted with undergraduate nursing students in the 2020 and 2021 cohort in December 2020 and 2021, respectively. The second survey was conducted with undergraduate nursing students in the 2020 and 2021 cohort in June 2023 and 2024, respectively. The training objectives, plans, modes and learning content of students in the two classes were completely consistent.

### 2.2 Population and sample

Cluster sampling was used to select two cohorts of all registered undergraduate students majoring in nursing from a medical university in Southeast of China for inclusion. After communication, all the students voluntarily agreed to participate in this study. The inclusion criteria were as follows: full-time undergraduate nursing students and students aged ≥18 years. Students who took a leave of absence, transferred, or joined the military midway through the study were excluded for various reasons. We invited 368 eligible nursing students to participate in the study, including 151 students from the 2020 cohort and 217 students from the 2021 cohort according to the enrolment number of the university.

In accordance with the recommended standards for structural equation modeling (SEM) and cross-lagged panel modeling (CLPM), each free parameter in the model requires a minimum of 10–20 observed samples (Kline, 2016). The cross-lagged model in this study comprises four variables (self-directed learning ability, collaborative ability, critical thinking, and problem-solving ability), encompassing the following primary pathways: four autoregressive paths (each variable predicting itself from t1 to t_2_), four cross-lagged paths (each variable influencing the development of another variable at t_2_), four covariance paths among variables at time point t1, and four residual variance estimates at time point t_2_, totaling ~16 free parameters. Based on a conservative estimation criterion, the minimum sample size should be: sample size ≥ 10 × d = 10 × 16 = 160. The study included 368 participants (*N* = 368), with each of the four variables measured at two time points, resulting in a total of 368 × 2 = 736 observed data points, which significantly exceeds the recommended standard. This ample sample size ensures the stability of model estimation and meets the requirements for statistical power.

### 2.3 Questionnaire

#### 2.3.1 General information survey for undergraduate nursing students

By referring to the relevant literature, a self-designed survey questionnaire was developed and used to understand the general information of the undergraduate nursing students, including the following: age, gender, ethnicity, whether the nursing major was the student's first choice at the time of the college entrance examination, and whether the student like the nursing major.

#### 2.3.2 Self-study ability

In this study, the “Scale for Assessing the Self-study Ability of Nursing Undergraduate Students,” developed by Lin Yi and Jiang Anli from the Second Military Medical University in 2004, was used to evaluate the self-study ability of the control group and the experimental group, respectively. The scale consists of 28 items, including subscales for self-management ability, information ability, and collaborative ability. There are 10 items for self-management ability, 11 items for information ability, and seven items for collaborative ability. A 5-point Likert scale is used for scoring, which includes responses of “completely agree,” “basically agree,” “average,” “basically disagree,” and “completely disagree.” The scores for forward-scored questions are 5, 4, 3, 2, and 1, whereas the scores for reverse-scored questions are the opposite: 1, 2, 3, 4, and 5, respectively. The score range is 28–140 points. The total score for self-study ability ranges from 28–140 points, that for self-management ability ranges from 10–50 points (1–10 questions), that for information ability ranges from 11–21 points, and that for collaborative ability ranges from 7–35 points (22–28 questions). The higher the score is, the stronger a student's self-study ability. This scale has good content validity and constructs validity, with a Cronbach's alpha coefficient of 0.86. Recent investigations have utilized this scale to assess reliability among nursing undergraduate populations, demonstrating a Cronbach's α coefficient of 0.845, which indicates robust internal consistency. These findings substantiate the instrument's appropriateness for evaluating self-directed learning competencies in contemporary nursing undergraduate cohorts (Zheng et al., 2023).

#### 2.3.3 Critical thinking ability

The Critical Thinking Disposition Inventory-Chinese Version (CTDI-CV) was translated and revised based on the California Critical Thinking Attitude Disposition Test. The scale is suitable for use among college students, graduate students, and adult professionals and was translated into Chinese by experts such as Professor Peng Meici from the School of Nursing at Hong Kong Polytechnic University in 2004. This scale is divided into seven dimensions, namely, seeking truth, open-minded thinking, analytical thinking, systematic thinking, self-confidence, inquisitiveness, and maturity, with 10 items in each dimension. Each item is scored based on the degree of approval: “strongly agree,” “completely agree,” “somewhat agree,” “generally agree,” “completely disagree,” and “strongly disagree.” The positive items are assigned “6 points, 5 points, 4 points, 3 points, 2 points, or 1 point,” whereas negative items are scored in reverse. The total score of the scale ranges from 70–420 points, with scores ≤ 210 points indicating negative evaluative thinking tendencies, score of 210–280 points indicating unclear evaluative thinking tendencies, scores ≥280 points indicating positive evaluative thinking tendencies, and scores ≥350 points indicating strong positive evaluative thinking tendencies. This scale is widely used in China and has high reliability and validity. The content validity index (CVI) is 0.89, the total Cronbach's alpha value is 0.90 (Peng et al., 2004).

#### 2.3.4 Cooperative ability

This study used the “Cooperative Ability Strength Scale” developed by Wang Bin and Li Furong from Central China Normal University in 2011 (Li, 2011). The scale has been empirically validated among nursing student populations, demonstrating robust psychometric properties with established reliability and validity indices (Wang et al., 2022). The scale consists of 42 questions divided into two aspects: cooperative awareness and cooperative skills. Cooperative awareness mainly includes three dimensions: cooperative cognition (Items 1–11), cooperative emotion (Items 12–17), and cooperative intention (Items 18–22). Cooperative skills include four dimensions: interpersonal interaction (Items 23–29), conflict management (Items 30–35), emotional control (Items 36–39), and organizational leadership (Items 40–42). The scale is scored on a 5-point Likert scale ranging from “completely disagree” to “completely agree.” The internal consistency reliability of this scale is 0.94, and the split half reliability is 0.87. The homogeneity reliability of the 7 factors ranges from 0.74 to 0.92, and the split half reliability ranges from 0.70 to 0.88.

#### 2.3.5 Problem-solving ability

Social problems include various issues that affect people, including but not limited to personal behavior problems, cognitive problems, interpersonal relationship problems, etc. The common social problems among college students in China include three categories: personal internal problems (47.53%), interpersonal relationship problems (40.66%), and learning problems (10.76%). Therefore, the revised D'Zurilla Social Problem Solving Questionnaire-Revised (SPSI-R), developed by Wang Fei and Liu Yan in 2009 in the context of Chinese culture, was adopted, with the aim of comprehensively and deeply exploring the problem-solving ability of undergraduate nursing students through a questionnaire. The Chinese version of the SPSI-R has good reliability and validity. The questionnaire consists of 32 items, including five factors: positive problem-solving tendency, negative problem-solving tendency, rational problem-solving tendency, avoidant problem-solving tendency, and impulsive negligent problem-solving tendency. The scores range from 1 “completely disagree” to 5 “completely agree.” The total scale and each factor have good internal consistency coefficients (Cronbach's coefficient of 0.85) and test-retest reliability (*r* = 0.50) (Schepers et al., 2023).

To evaluate the psychometric properties of these measurement instruments, we performed a test-retest reliability analysis involving thirty participants. The intraclass correlation coefficients (ICCs) for the Self-study Ability Scale, Critical Thinking Ability Scale, Cooperative Ability Scale, and Problem-solving Ability Scale were determined to be 0.880, 0.885, 0.834, and 0.814, respectively. These reliability coefficients, all exceeding the conventional threshold of 0.70, demonstrate satisfactory temporal stability and indicate that the scales exhibit appropriate measurement consistency for the study population.

### 2.4 Survey

The timing of questionnaire distribution, which was scheduled during students' free time after exams, reducing their time pressure. Before the questionnaire was distributed, the researcher used unified guiding language to explain the questionnaire to the research participants, and after the students provided informed consent, the questionnaire was distributed on site. The completion time for the questionnaire was 15 min. The students were asked to complete the questionnaire carefully according to their actual situation. The use of small gifts as incentives during questionnaire collection, which was solely intended to enhance enthusiasm without compromising the principle of voluntariness. To ensure confidentiality, the questionnaire was anonymous; the purpose of the questionnaire, which was only for the study and not for any other purpose, was explained to the research participants. To ensure the validity of the questionnaire, explanations were provided for questions that the research participants did not understand, and missing items were promptly supplemented. All survey scales were distributed onsite by the researchers themselves for verification and collection. During the questionnaire collection process, the researcher conducted on-site inspections of the completion status. In cases of missing data, on-site guidance and supervision were provided, and the research subjects were requested to complete the missing information. After collecting the questionnaires, a second check was performed to ensure the completeness and validity of the questionnaires, excluding those with excessive missing data or high repetition rates in responses.

### 2.5 Data analysis

Initial descriptive statistical analyses were conducted using SPSS version 27.0 to characterize the fundamental distributional properties of each variable. Subsequently, bivariate correlation analyses were performed to examine the interrelationships between variables, with Pearson's correlation coefficients and corresponding *p*-values calculated at both temporal measurement points. For the longitudinal analysis, a cross-lagged panel model was constructed and evaluated using the Lavaan package in R software to assess both self-lagged and cross-lagged effects among the study variables.

Time series data encompassing self-study, collaborative, critical thinking, and problem-solving abilities were employed in this study. The dataset comprises observations at two time points, with data recorded as independent and dependent variables at each time point. The fundamental principle of cross-lagged models is to analyze the dynamic relationships between variables by simultaneously considering their observed values across multiple time points. Within the framework of structural equation modeling (SEM), these models enable researchers to assess both autoregressive and cross-lagged effects simultaneously, thus exploring potential causal relationships among variables. This framework has been widely used to examine longitudinal and bidirectional associations between constructs in sociological and psychological research (Tak et al., 2017; Wang et al., 2020).

Suppose that we have four variables, *X*1, *X*2, *X*3, and *X*4, representing self-study ability, collaborative ability, critical thinking ability, and problem-solving ability, respectively. At time points *t*1 and *t*2, the values of these variables are denoted as *Xi*, 1 and *Xi, t*2 (where *i* = 1, 2, 3, 4). The cross-lagged model can be represented by the following equation:


$$\begin{array}{l} Xi,t2=\beta iXi,t1+\sum j\neq i\gamma ijXj,t1+\;\epsilon i \end{array}$$

β*i* denotes the self-lagged effect coefficient for variable *Xi*, indicating the stability of this variable over time.

γ*ij* signifies the cross-lagged effect coefficient for variable *Xj* relative to *Xi* across different time points, reflecting the interaction between the variables across time.

ϵ*i* is the residual term, indicating the unexplained portion of the model.

In this study, the specific model equation is as follows:


$$\begin{array}{l} \text{Self}-\text{studyt}2=\beta \text{Self}-\text{study }\times \text{ Self}-\text{studyt}1+\gamma \text{Collab}\\ \;\times \;Collab\text{t}1+\;\epsilon Self-study\\ \text{ Problemt}2=\beta \mathrm{Problem}\;\times \;\mathrm{Problem}\text{t}1+\;\epsilon \text{Problem}\\ \text{ Collabt}2=\beta \text{Collab }\times \text{ Collabt}1+\gamma \text{Problem }\times \text{ Problemt}1\\ \;+\;\epsilon Collab\\ \text{ Criticalt}2=\beta \text{Critical }\times \text{ Criticalt}1+\gamma \text{Self}\\ \;-study\;\times \;Self-study\text{t}1+\;\epsilon Critical \end{array}$$

These equations describe how the value of each variable at time *t*2 is influenced by its value at time *t*1 and by other variables.

The self-lagged effect coefficient β*i* represents the stability of the variable across different time points. A larger β*i* indicates stronger temporal consistency of the variable, implying that the state of the variable at time *t*1 provides a stronger prediction for its state at time *t*2.

#### 2.4.1 Cross-lagged effect (γ*ij*)

The cross-lagged effect coefficient γ*ij* reflects the influence of one variable at an earlier time point on another variable at a later time point. The significance of this coefficient is that it can be used to infer potential causal relationships between variables. For instance, a significant γ Collab indicates that collaborative ability at time *t*1 significantly influences self-study ability at time *t*2. The goodness of fit of the Cross-Lagged Panel Model (CLPM) was comprehensively evaluated via the comparative fit index (CFI), Tucker-Lewis index (TLI), and root mean square error of approximation (RMSEA). A CFI ≥ 0.9, a TLI ≥ 0.9, and an RMSEA ≤ 0.06 indicate that a model has a good fit, and a CFI and TLI ≥ 0.80 and an RMSEA ≤ 0.08 indicate acceptable fit (Zhang et al., 2020).

### 2.5 Ethical considerations

In November 2020, prior to the initiation of this investigation, we submitted an ethical review application to the Institutional Review Board (IRB) of the affiliated university. Following comprehensive evaluation, the IRB granted ethical exemption for this study, categorizing it as a preliminary educational reform investigation that presents no foreseeable risks to participants. The committee determined that the study met the institutional criteria for exemption from full ethical review, as it involved minimal risk and did not compromise participant welfare or confidentiality. All research was performed in accordance with relevant guidelines. All the data were anonymized for coding and analysis to safeguard participant privacy. Prior to the survey, the participants were informed of the voluntary and confidential principles of the study and that not all the data files contained sensitive information about the participants. All research participants provided informed consent prior to their involvement in this study.

## 3 Results

### 3.1 Summary of the study sample

At the first time point, 368 research subjects participated in the survey. At the second time point, all research subjects participated in the survey again. The general information for the 2020 and 2021 cohorts is shown in Table 1. There was no statistically significant difference between the two groups except for ethnicity and occupational status (*P* > 0.05), whereas all other differences were statistically significant (*P* < 0.05).

**Table 1:** General information of the two cohorts [Person, percentage (%)].

**Variable**	**2020 cohort (*n* = 151)**	**2021 cohort (*n* = 217)**	***χ^2^***	***P***
**Sex**			5.671	**0.017**
Male	38 (25.2)	33 (15.2)		
Female	113 (74.8)	184 (84.8)		
**Ethnicity**			0.083	0.773
Han	135 (89.4)	196 (90.3)		
Other	16 (10.6)	21 (9.7)		
**Place of origin**			21.391	**< 0.001**
Rural	104 (68.9)	115 (53.0)		
Urban	38 (25.2)	69 (31.8)		
Urban–rural fringe	9 (6.0)	33 (15.2)		
**Nursing major is first choice**			28.334	**< 0.001**
Yes	17 (11.3)	78 (35.9)		
No	134 (88.7)	139 (64.1)		
**Reason for applying**			21.391	**< 0.001**
Voluntary choice	45 (29.8)	109 (50.2)		
Parental choice	15 (9.9)	28 (12.9)		
Teacher's choice	1 (0.7)	3 (1.4)		
Major adjustment	90 (59.6)	77 (35.5)		
**Serves as a student leader**			3.931	**0.047**
Yes	79 (52.3)	136 (62.7)		
No	72 (47.7)	81 (37.3)		
**Level of major preference**			15.825	**< 0.001**
Very much	54 (35.8)	38 (17.5)		
Neutral	88 (58.3)	163 (75.1)		
Dislike	9 (6.0)	16 (7.4)		
**Engaged in this profession**			0.096	0.953
Yes	81 (53.6)	118 (54.4)		
No	8 (5.3)	10 (4.6)		
Unsure	62 (41.1)	89 (41.0)		

*X*^2^, chi-square test. The bold font in the table represents *p* < 0.05.

The scores for self-study, critical thinking, cooperation, and problem-solving abilities were subjected to normality tests. Except for the scores for self-study ability of the 2020 and 2021 cohorts in the first year, which conformed to a normal distribution (*P* > 0.05), all the other scores did not (*P* < 0.05). Consequently, non-parametric tests were employed, as detailed in Table 2. The scores for self-study, critical thinking, and collaborative abilities of the 2020 cohort in the third year were significantly higher than those in the first year (*P* < 0.05). Similarly, the scores for self-study, critical thinking, cooperative, and problem-solving abilities of the 2021 cohort in the third year were significantly higher than those in the first year (*P* < 0.05). Significant differences were observed in the scores for self-study, critical thinking, and collaborative abilities between the two cohorts in the first year (*P* < 0.05). Significant differences were also noted in the scores for self-study and collaborative abilities between the two cohorts in the junior year (*P* < 0.05).

**Table 2:** Comparison of the four learning abilities between the two cohorts in the 2 years [score, M (P25, P75)].

**Variable**	**2020 cohort (*****n*** = **151)**		**2021 cohort (*****n*** = **217)**		***Z*_1_**	***P_1_***	***Z*_2_**	***P_2_***	***Z*_3_**	***P_3_***	***Z*_4_**	***P_4_***
	**Freshmen**	**Juniors**	**Freshmen**	**Juniors**								
Self-study ability	78.00 (75.00, 82.00)	95.00 (89.00, 100.00)	76.00 (72.00, 80.00)	91.00 (87.00, 94.50)	7.250	**< 0.001**	−16.726	**< 0.001**	−3.279	**0.001**	−5.189	**< 0.001**
Critical thinking ability	251.00 (240.00, 274.00)	264.00 (247.00, 286.00)	256.00 (245.00, 279.00)	266.00 (250.00, 290.00)	1.611	**0.011**	−2.974	**0.003**	−2.017	**0.044**	−1.034	0.301
Cooperative ability	146.00 (137.00, 153.00)	155.00 (148.00, 164.00)	106.00 (98.50, 114.00)	146.00 (139.00, 154.00)	3.453	**< 0.001**	−16.371	**< 0.001**	−14.034	**< 0.001**	−6.045	**< 0.001**
Problem-solving ability	103.00 (98.00, 108.00)	105.00 (98.00, 112.00)	103.00 (96.00, 108.00)	105.00 (98.00, 112.00)	1.151	0.141	−2.152	**0.031**	−0.263	0.792	−0.551	0.582

Z_1_ and P_1_ are comparisons between freshmen and juniors in the 2020 cohort; Z_2_ and P_2_ are comparisons between freshmen and juniors in the 2021 cohort; Z_3_ and P_3_ are the two groups of freshmen for comparison; Z_4_ and P_4_ are the two groups of juniors for comparison. The bold font in the table represents *p* < 0.05.

### 3.2 Correlation analysis results

**Figure 1:**
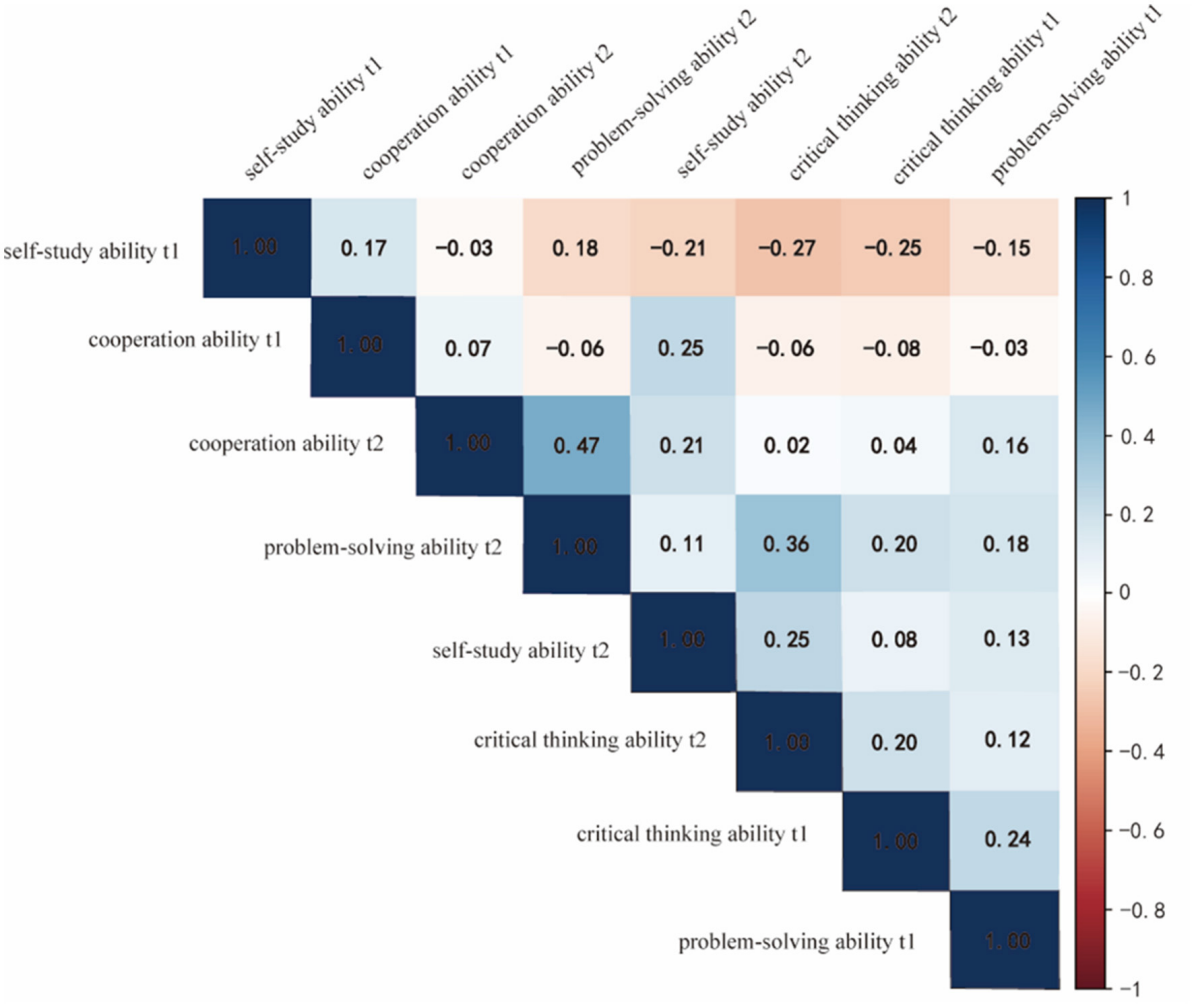
Results of the correlation analysis of self-study, cooperation, critical thinking, and problem-solving abilities.

Spearman correlation analysis revealed significant correlations among certain variables. Specifically, problem-solving ability was correlated with other variables across different time points. Problem-solving ability at the first time point was correlated with self-study, cooperative, critical thinking, and problem-solving abilities at the first time point (*P* < 0.05). Problem-solving ability at the second time point was correlated with self-study ability at the first time point, critical thinking and problem-solving abilities at the first time point, and self-study and collaborative abilities at the second time point (*P* < 0.05). This suggests potential interactions among these abilities. Additionally, collaborative ability at the first time point was correlated with self-study ability (*P* < 0.05). Self-study ability at the first time point was negatively correlated with critical thinking and problem-solving abilities at both time points and self-study ability at the second time point (*P* < 0.05) (Table 3; Figure 1).

**Table 3:** Results of the correlation analysis of the four variables at the two time points.

**Variable**	**Self-study ability-1**	**Collaborative ability-1**	**Problem-solving ability-1**	**Critical thinking ability-1**
Self-study ability-1	1	0.166^**^	−0.148^**^	−0.248^**^
Collaborative ability-1	0.166^**^	1	−0.025	−0.080
Problem-solving ability-1	−0.148^**^	−0.025	1	0.243^**^
Critical thinking ability-1	−0.248^**^	−0.080	0.243^**^	1
Self-study ability-2	−0.215^**^	0.247^**^	0.135^**^	0.083
Collaborative ability-2	−0.025	0.070	0.158^**^	0.044
Problem-solving ability-2	−0.185^**^	−0.065	0.179^**^	0.202^**^
Critical thinking ability-2	0.274^**^	−0.057	0.119^*^	0.200^**^

^**^P < 0.01; ^*^P < 0.05.

### 3.3 Cross-lagged model results

**Figure 2:**
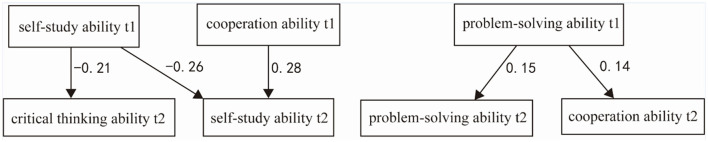
Cross-lagged model results for self-study, cooperative, critical thinking, and problem-solving abilities for the total sample.

The path coefficients and their significance levels revealed intriguing causal relationships. First, self-study ability had a significant and negative predictive effect from Time 1 to Time 2 (β = −0.342, *p* < 0.001). Second, collaborative ability had a significant and positive effect on self-study ability from Time 1 to Time 2 (γ = 0.088, *p* < 0.001). Third, problem-solving ability had a significant and positive self-predictive effect from Time 1 to Time 2 (β = 0.176, *p* < 0.01). Additionally, problem-solving ability had a significant and positive effect on collaborative ability from Time 1 to Time 2 (γ = 0.225, *p* < 0.01). Finally, self-study ability had a significant and negative effect on critical thinking ability from Time 1 to Time 2 (γ = −1.149, *p* < 0.001). We ultimately only selected paths that exhibited significant effects and could converge to establish the model. The primary reason is that other paths exhibited weak correlations, and their effects remained insignificant even when they were gradually incorporated into the model. Therefore, we ultimately did not include these paths (Table 4; Figure 2).

**Table 4:** Standardized coefficients of self-study, cooperation, critical thinking, and problem-solving abilities for the overall sample on the basis of a cross-lagged panel model.

**Parameter**	**β**	**SE**	***P***	**95% confidence interval**	
				**Lower**	**Upper**
**Stability paths**					
Self-study ability-1 → Self-study ability-2	−0.264	0.063	< 0.001	−0.466	−0.219
Problem-solving ability-1 → Problem-solving ability-2	0.151	0.057	0.002	0.064	0.287
**Cross-lagged effects**					
Self-study ability-1 → Critical thinking ability-2	−0.212	0.257	< 0.001	−1.653	−0.645
Cooperation ability-1 → Self-study ability-2	0.275	0.015	< 0.001	0.059	0.118
Problem-solving ability-1 → Cooperation ability-2	0.141	0.081	0.006	0.066	0.384

### 3.4 Sensitivity analysis results

Sensitivity analysis was performed to evaluate the robustness of the study. The model demonstrated acceptable fit (CFI = 0.943, TLI = 0.854, RMSEA = 0.078).

## 4 Discussion

First, an unexpected finding of this study was that the self-study ability of junior nursing students negatively affected their self-study and critical thinking abilities as they progressed to more advanced learning stages. The results of the correlation analysis revealed that self-study ability at the first time point was negatively correlated with critical thinking ability and problem-solving ability at both time points and self-study ability at the second time point. The results of the present study demonstrate a divergence from previous research findings. Several empirical investigations have consistently shown that both cognitive abilities and self-directed learning competencies exhibit significant positive predictive relationships with academic performance in higher education settings (Cassady et al., 2022; Zheng et al., 2021). Therefore, within the Chinese educational context, primary and secondary school students may focus primarily on exam-oriented education, leading to poor learning adaptation, with the result that lower-grade nursing students continue to adopt their original learning and thinking patterns after entering university. Although students demonstrate a strong ability to learn from classroom instruction and textbooks, they lack independent thinking and a questioning spirit and struggle with knowledge application (Chen et al., 2018). As they progress to higher-level professional courses, their traditional self-study methods prove inadequate in addressing the challenges posed by clinical applications (Sun et al., 2024). In China, blended learning is extensively employed in various nursing courses, including internal medicine nursing, surgical nursing, geriatric nursing, and obstetrics and gynecology nursing courses. This presents significant challenges for nursing students in terms of critical thinking and problem-solving skills (Kong et al., 2022; Zhou et al., 2023). Clearly, exam-focused self-study ability is not applicable to the study of professional courses in later academic years, which may be the primary cause of their adverse effects. Therefore, it is recommended that teachers enhance students' abilities to apply knowledge and facilitate a shift from traditional learning methods and mindsets by using innovating teaching methods during the foundational medical phase for nursing students. This approach facilitates the transformation of students' learning methodologies and cognitive frameworks, thereby enhancing their autonomous learning capabilities in both specialized academic curricula and clinical nursing practice. Consequently, this pedagogical strategy contributes to the progressive development of students' critical thinking competencies (Hwang and Oh, 2021).

Second, the problem-solving skills of junior nursing students positively affected their collaborative and problem-solving abilities in their senior year. Enhanced problem-solving skills lead to better application of professional knowledge. This result is similar to other research findings (Lee et al., 2025; Selçuk Tosun et al., 2025). Only when students grasp how to apply knowledge do they understand how to proactively seek knowledge rather than solely focusing on exam preparation (Huang et al., 2023). This progressive enhancement of self-study and collaborative skills in obtaining and applying knowledge creates a virtuous cycle in terms of problem-solving abilities (Cheng et al., 2024).

Third, the results of this study also indicated a close relationship between cooperative ability and self-study ability. This result is similar to other research finding. Research evidence indicates that blended learning environments necessitate student engagement in collaborative activities, task allocation, autonomous development of learning strategies, goal setting, and collective problem-solving through interactive discussions and knowledge exchange (Padugupati et al., 2021). This learning method enables students to demonstrate initiative and creativity, fosters students' self-study ability, enhances students' problem-solving skills, and improves learning outcomes. This is instrumental in fostering students' lifelong learning abilities. This is especially crucial for nursing students, as nursing work requires teamwork and a division of labor to assist patients in achieving their care goals. Furthermore, as clinical knowledge and concepts evolve rapidly, nurses must engage in lifelong learning to excel in their profession (Deng, 2022). With the advancement of the internet, students can access a wealth of educational resources and information online, utilizing digital technologies to proactively learn and obtain knowledge, which also creates more opportunities for collaboration with others. Students can use online collaboration tools and platforms to work with others on various tasks and projects, thus further improving their collaborative skills (Chen et al., 2020). This finding also suggests that during the learning process, teachers should utilize various activities, such as knowledge expansion, academic competitions, professional practices, innovation contests, and volunteer work, to encourage students to proactively seek, expand, and apply knowledge through the internet. This approach aims to enhance students' collaborative skills and, in turn, strengthen their autonomous learning abilities.

Fourth, the correlation analysis results showed that critical thinking ability stands out as relatively independent, its beneficial impact on the development of critical thinking and problem-solving abilities cannot be overlooked. Critical thinking is the ability to engage in the in-depth analysis, evaluation, and reasoning of information (Liu et al., 2020). It helps individuals capture the essence of issues and avoid being misled by superficial appearances (Huang et al., 2020). In making judgements about various matters, it is essential for learners to employ problem-solving skills, which aid them in deeply understanding and assimilating knowledge, distinguishing between surface phenomena and underlying causes, pinpointing the essence of problems, and making accurate judgements about knowledge (Liang et al., 2019). The reason for the relative independence of critical thinking ability is that its development is a holistic process that requires the capacity for independent thought, a spirit of inquiry and discernment, judgement founded on rigorous inference, extensive cognitive training and knowledge reserves, multiple methods and stages of thinking, a spirit of critique and a capacity for rational thought, along with qualities such as curiosity, imagination, and a desire for knowledge. Consequently, any single aspect of learning ability is inadequate to significantly influence critical thinking ability, and further comprehensive assessment and analysis are necessary for research into the effect of critical thinking ability on nursing students. Meanwhile, based on the above research conclusions. Although the self-study ability of lower-grade nursing students is positively correlated with their collaborative ability, it is negatively correlated with critical thinking. Moreover, there is no correlation between collaborative ability and critical thinking ability. This further suggests that the collaborative ability of lower-grade nursing students is limited to passing exams rather than applying professional knowledge to collaboratively solve professional problems.

This study reflects the cultural specificity of Chinese education. Within the framework of traditional Chinese educational paradigms, the foundational education system predominantly adopts a “teacher-centric, knowledge-transmission” pedagogical approach. Nursing students emerging from this cultural milieu frequently demonstrate distinct behavioral patterns: (1) pronounced dependence on explicit instructional guidance, with limited capacity for autonomous learning strategy formulation; (2) predisposition toward passive knowledge acquisition, accompanied by underdeveloped critical thinking and problem identification skills; (3) a reductionist conceptualization of learning as mere memorization and examination preparation. As these students progress to advanced academic levels and encounter scenarios necessitating self-directed learning trajectory planning and proactive clinical inquiry, their entrenched learning modalities present significant impediments. Consequently, in the development of student training curricula, academic institutions should implement structured support mechanisms (including phased case repositories and reflective practice instruments) to mitigate competency deficits, while progressively employing empowerment-based instructional strategies to facilitate the transition from “passive compliance” to “strategic self-regulated learning.”

## 5 Conclusion

Undergraduate nursing students' self-study, collaborative and problem-solving abilities are interrelated, whereas their critical thinking ability is distinct. Contrary to our initial hypotheses, self-directed learning ability during the early academic years demonstrated a significant negative correlation with both self-directed learning capacity and critical thinking skills in subsequent academic years. This unexpected finding appears to be closely associated with the specific context of traditional Chinese pedagogical approaches, which may influence the developmental trajectory of these cognitive skills. The factors influencing these abilities merit thorough analysis and investigation. Beginning in the early years of study, educators should revamp teaching methods and models, leveraging avenues such as intellectual exploration, academic contests, practical training, innovation fairs, and volunteer work to assist students in unlearning conventional learning and thought processes. Teachers should aim to foster collaborative problem-solving skills by applying knowledge, thereby augmenting students' overall problem-solving ability.

## 6 Limitations

This study has several limitations. First, only the data of undergraduate nursing students from one provincial medical university and one course were evaluated in this study, and data were not collected from students in other levels of medical colleges and other professional courses. Future research needs to expand the sample source range to make the data more representative. Second, data were collected at only two time points; we were unable to collect data at more time points, such as after the internship. Therefore, in the future, it is necessary to continue collected the data of nursing students during their internships and even after work to dynamically evaluate the changes in their critical thinking, collaborative ability, and self-study abilities.

## Data Availability

The raw data supporting the conclusions of this article will be made available by the authors, without undue reservation.
